# Tryptophan was metabolized into beneficial metabolites against coronary heart disease or prevented from producing harmful metabolites by the *in vitro* drug screening model based on *Clostridium sporogenes*

**DOI:** 10.3389/fmicb.2022.1013973

**Published:** 2022-11-16

**Authors:** XiaoXue Tian, Yuanyuan Wu, Cheng Duan, Xiaohong Zhou, Yong Li, Jiabin Zheng, Weihua Lai, Shuyao Zhang, Lixiang Cao, Shilong Zhong

**Affiliations:** ^1^Guangdong Cardiovascular Institute, Guangdong Provincial People’s Hospital, Guangdong Academy of Medical Sciences, Guangzhou, Guangdong, China; ^2^School of Pharmaceutical Sciences, Southern Medical University, Guangzhou, Guangdong, China; ^3^Department of Pharmacy, Guangdong Provincial People’s Hospital, Guangdong Academy of Medical Sciences, Guangzhou, Guangdong, China; ^4^Greater Bay Area Institute of Precision Medicine (Guangzhou), Guangzhou, Guangdong, China; ^5^The Second School of Clinical Medicine, Southern Medical University, Guangzhou, Guangdong, China; ^6^Department of Pharmacy, Guangzhou Red Cross Hospital (Guangzhou Red Cross Hospital of Jinan University), Guangdong, China; ^7^School of Life Sciences, Sun Yat-sen University, Guangzhou, Guangdong, China

**Keywords:** *in vitro* drug screening model, tryptophan-derived metabolites, *Clostridium sporogenes*, indole-3-butyric acid, coronary heart disease

## Abstract

In our previous study of 2,130 Chinese patients with coronary heart disease (CHD), we found that tryptophan (TRP) metabolites contributed to elevated risks of death. Many TRP-derived metabolites require the participation of intestinal bacteria to produce, and they play an important role in the pathogenesis of metabolic diseases such as CHD. So it is necessary to metabolize TRP into beneficial metabolites against CHD or prevent the production of harmful metabolites through external intervention. Indole-3-butyric acid (IBA) may be a key point of gut microbiota that causes TRP metabolism disorder and affects major adverse cardiovascular events in CHD. Therefore, this study aimed to develop a method based on *in vitro* culture bacteria to evaluate the effects of IBA on specific microbial metabolites quickly. We detected the concentrations of TRP and its metabolites in 11 bacterial strains isolated from feces using liquid chromatography–mass spectrometry, and selected *Clostridium sporogenes* as the model strain. Then, IBA was used in our model to explore its effect on TRP metabolism. Results demonstrated that the optimal culture conditions of *C. sporogenes* were as follows: initial pH, 6.8; culture temperature, 37°C; and inoculum amount, 2%. Furthermore, we found that IBA increases the production of TRP and 5-HIAA by intervening TRP metabolism, and inhibits the production of KYNA. This new bacteria-specific *in vitro* model provides a flexible, reproducible, and cost-effective tool for identifying harmful agents that can decrease the levels of beneficial TRP metabolites. It will be helpful for researchers when developing innovative strategies for studying gut microbiota.

## Introduction

In our previously prospective study with 2,130 Chinese patients with CHD, we have identified and validated TRP metabolites were causally contributed to left ventricular remodeling or elevated risks of death or major adverse cardiovascular events (MACE) signatures through widely-targeted metabolomics (The results have been accepted by *Cell & Bioscience*). The gut microbiota is a large and diverse community of microbes that interact with the host in the body and has an important influence on the key physiological functions of the host, such as metabolism and nutrition, immune system, and brain activity (Zhang et al., 2021). The disequilibrium of intestinal microbiota and related metabolites has attracted more attention for their potential association with CHD (Jie et al., 2017; Liu et al., 2021a). TRP metabolites are directly or indirectly regulated by intestinal microbiota. Studies have shown that the intestinal microbiota can affect cardiovascular diseases by participating in the TRP metabolic pathways (Gheorghe et al., 2019; Purton et al., 2021). The aryl hydrocarbon receptor (AHR) is a ligand-activated transcription factor that is closely associated with inflammation, oxidative stress, lipid infiltration, and atherosclerosis. Numerous TRP metabolites, which can regulate the intestinal barrier function through AHR, are ligands of AHR (Liu et al., 2021b). In preclinical and clinical settings, metabolic disorders are characterized by a reduced ability of microbiota to metabolize TRP as an AHR agonist. TRP and several bacterial TRP metabolites, such as indole, indole-3-propionic acid (IPA), and 3-indole-acetic acid (IAA), promote the production of the proinflammatory cytokine IL-22 by activating AHR (Taleb, 2019). In such cases, treatment with AHR agonists or bacteria that naturally produce AHR ligands, such as *Lactobacillus reuteri*, can reverse metabolic dysfunction (Natividad et al., 2018).

The TRP metabolic pathway has been found in some human intestinal microorganisms, such as *Clostridium sporogenes*, which is the most famous IPA producer (Zhang and Davies, 2016; Dodd et al., 2017). *C. sporogenes* is a gram-positive, periflagellate, strictly anaerobic clostridium bacterium that is commonly found in the soil and intestines and feces of humans and animals (Poehlein et al., 2015). At present, *C. sporogenes* is often considered as a solid tumor targeting vector with excellent targeting performance and high safety (Guo et al., 2019). In addition, *C. sporogenes* has been known as a robust producer of highly abundant small molecules for a long time, and it is a commensal or mutualist (i.e., neither a pathogen nor a pathobiont; Dodd et al., 2017) in the gut microbiome. The objectives of this study were to establish an *in vitro* model that can detect the changes of TRP metabolites with bacteria. Thus, the model bacteria must be able to metabolize TRP in large quantities and produce important TRP metabolites, such as IPA, 5-HT, and 5-hydroxyindole-3-acetic acid (5-HIAA). Notably, not all bacteria can metabolize TRP. Thus, we screened a set of bacterial isolates and performed metabolic profiling experiments to determine the set of highly abundant TRP-derived metabolites that can be produced by the most suitable strain *in vitro* systematically.

The efficacy of various drugs can be affected by gut microbes and the production of related metabolites. Evidence shows that simvastatin affects lipid-lowering function by changing the abundance and diversity of intestinal microbiota and inducing changes in the expression profile of bile acids, which are metabolites of intestinal microbiota. This effect is weakened in antibiotic-treated mice, suggesting that the lipid-lowering effect of simvastatin partly depends on intestinal microbiota (He et al., 2017). According to another study, resveratrol can inhibit the production of trimethylamine by remodeling the intestinal microbiota to alleviate the atherosclerotic phenotype of ApoE^−/−^ mice induced by trimethylamine oxide (Chen et al., 2016). In addition, oral propionic acid or targeting the intestinal microbiota to promote propionic acid production may help reduce atherosclerotic lesions (Bartolomaeus et al., 2019). Convincing evidence also demonstrates that metformin alters the composition of the intestinal microbiota (e.g., reduces the abundance of *Enterobacteriaceae*) and directly affects the intestinal bacterial metabolism (e.g., increases butyrate production; Forslund et al., 2015; Pollak, 2017; Wu et al., 2017). Similarly, the orally administered cardiac drug digoxin is changed by certain gut bacteria, reducing its effectiveness (Haiser et al., 2013). Therefore, intestinal bacteria-derived metabolites have significant associations with drug action, which suggests that the *in vitro* culture of intestinal microorganisms has a broad application prospect.

Decreased TRP concentrations were found in a significant proportion of CHD patients (Wirleitner et al., 2003; Murr et al., 2015; Liu et al., 2017). Some studies have reported that the disease status of CHD can affect the abnormal metabolism of TRP (Song et al., 2017; Platten et al., 2019; Bakhta et al., 2020).Yu et al. reported that urinary TRP maybe associated with the risk of CHD among Chinese adults (Yoon et al., 2020). Kobchai et al. reported that the possible use of blood TRP metabolites as biomarkers for CHD in sudden unexpected death (Santisukwongchote et al., 2019). Moreover, many studies have reported that the increased levels of kynurenine (KYN) and kynurenic acid (KYNA), a main metabolite of TRP, was related to cardiovascular events and mortality, the KYN pathway can regulate vascular inflammation and atherosclerosis in a direct or indirect mode (Ray et al., 2007; Zuo et al., 2016; Verheyen et al., 2017; Yu et al., 2017). Besides, the ratio of KYN/TRP may a sensitive pointer of rigorous coronary events for individuals displaying no history of CHD, also can be used to predict dangerous coronary events even use to determining all-cause mortality for individuals suffering from CHD (Pedersen et al., 2011; Sulo et al., 2013). Additionally, a new review of more than 20 clinical studies also shown that the change of other TRP metabolites/TRP ratio also predict the significant CHD, so TRP pathway metabolites may as potential clinical biomarkers in CHD (Gáspár et al., 2021). Our previous plasma metabolome and genome-wide association studies also found that IPA may have a potential protective effect on patients with CHD (Wang et al., 2021). The conclusion was supported by other study that plasma tryptophan and IPA levels are significantly associated with decreased risk of cardiovascular and all-cause mortality in CHD patients (Li et al., 2022). In conclusion, we will establish an *in vitro* drug screening model to increase TRP and decrease KYN and KYNA or the ratio of KYN/TRP through drug intervention. In order to achieve the intervention of TRP metabolic pathway, increase beneficial metabolites or prevent its generation of harmful metabolites to CHD.

There may be a potential association between indole-3-butyric acid (IBA) and MACE from CHD. IBA is an auxin synthesized by plants, and plants can oxidize TRP to produce indole-3-pyruvate, which can further produce IPA and IBA (Damodaran and Strader, 2019). IBA may be ingested by the human body through food, absorbed into the blood, and then excreted through the urinary system. It is generally believed that auxin does not affect the normal physiological functions of the human body. Strikingly, IBA was a risk factor for MACE in patients with CHD in our previous univariate and multivariate analyses, which involved plasma metabolomics studies in single-center and multicenter validation cohorts (Zhu et al., 2018). Some studies also show that IBA causes the decrease of frusemide protein binding in acute renal failure plasma (Ikeda et al., 1984). Thus, IBA was used in our model to explore its effect on TRP metabolism.

In this study, orthogonal experiment design and liquid chromatography–mass spectrometry (LC–MS/MS) technology were combined to establish a new bacterial *in vitro* metabolism model. *C. sporogenes* was chosen as the model because of its remarkable properties. Our results demonstrated that IBA had a significant impact on bacteria-mediated TRP metabolism. Our model is expected to become an alternative strategy for finding a new drug for the treatment and prevention of CHD by interfering with microbiota to regulate TRP metabolism.

## Materials and methods

### Chemicals

Reinforced Clostridium Medium (RCM) was purchased from Qingdao Hope Bio-Technology Co., Ltd. (Supplementary Table S1). Acetonitrile (CAS.75-05-8, ≥99.9% purity), formic acid (CAS.64-18-6, ≥99.0% purity), ammonium acetate (CAS 631-61-8, ≥99.0% purity), methanol (CAS.67-56-1, ≥99.0% purity), dimethyl sulfoxide (DMSO, CAS.67-68-5, ≥99.9% purity), TRP (CAS.73-22-3, 98% purity), 3-hydroxykynurenine (3-HK, CAS.484-78-6, ≥99.0% purity), xanthurenic acid (XA, CAS.59-00-7, 96% purity), and IBA (CAS.133-32-4, ≥99.0% purity) were obtained from Sigma-Aldrich (United States). 3-Hydroxyanthranilic acid (3-HAA, CAS.548-93-6, ≥97.0% purity), 5-HIAA (CAS.54-16-0, 98% purity), 5-hydroxytryptamine (5-HT, CAS.50-67-9, ≥98% purity), IAA (CAS.87-51-4, ≥99.0% purity), IPA (CAS.830-96-6, ≥98% purity), KYNA (CAS.492-27-3, 97% purity), KYN (CAS.13441-51-5, 98% purity), and 5-hydroxytryptamine-d4 (5-HT-d4, CAS.58264-95-2, 98% purity) were purchased from Aladdin (China).

### Bacterial strains

A consensus has been reached that differences exist in the gut microbiotas of patients with CHD, and the gut microbiota is involved in mediating basic metabolic processes induce the development of atherosclerosis and CHD (Liu et al., 2020). According to two reviews of intestinal microbiota in patients with CHD and our laboratory platform, 11 strains of bacteria were isolated from the feces of patients with CHD, which were used to construct candidate strains for drug screening models (Kazemian et al., 2020; Kim et al., 2022). Including *Enterobacter hormaechei*, *Enterobacter cloacae*, *Klebsiella pneumoniae*, *Escherichia fergusonii*, *Weissella confusa*, *Klebsiella michiganensis*, *Shigella flexneri*, *Shigella sonnei*, *Klebsiella singaporensis*, and *Proteus columbae* were isolated and identified from the feces of patients with CHD and identified by Qingke Biotechnology Co., Ltd. (Beijing, China) based on 16S rDNA sequence analysis.

The types of bacteria, required medium, and gaseous environment are provided in Supplementary Tables S2–S6. The bacteria were isolated in a 60 mm round dish containing a solid medium and cultured in a 6 × 30 mm bacterial culture tube containing the appropriate liquid medium (without agar). They were then placed in incubators under their specific culture conditions. The *C. sporogenes* used in our experiment was CMCC 64941, which was obtained from The Microbial Strain Preservation Center of Guangdong Province, China. The strain was then identified as *C. sporogenes* by Qingke Biotechnology Co., Ltd. (Beijing, China) based on 16S rDNA sequence analysis. The isolated strain shared 100% sequence similarity to the *C. sporogenes* strain ATCC15579. The bacteria used were approved by the Biosafety Committee of Guangdong Provincial People’s Hospital.

### Screening of model bacteria

**Figure 1:**
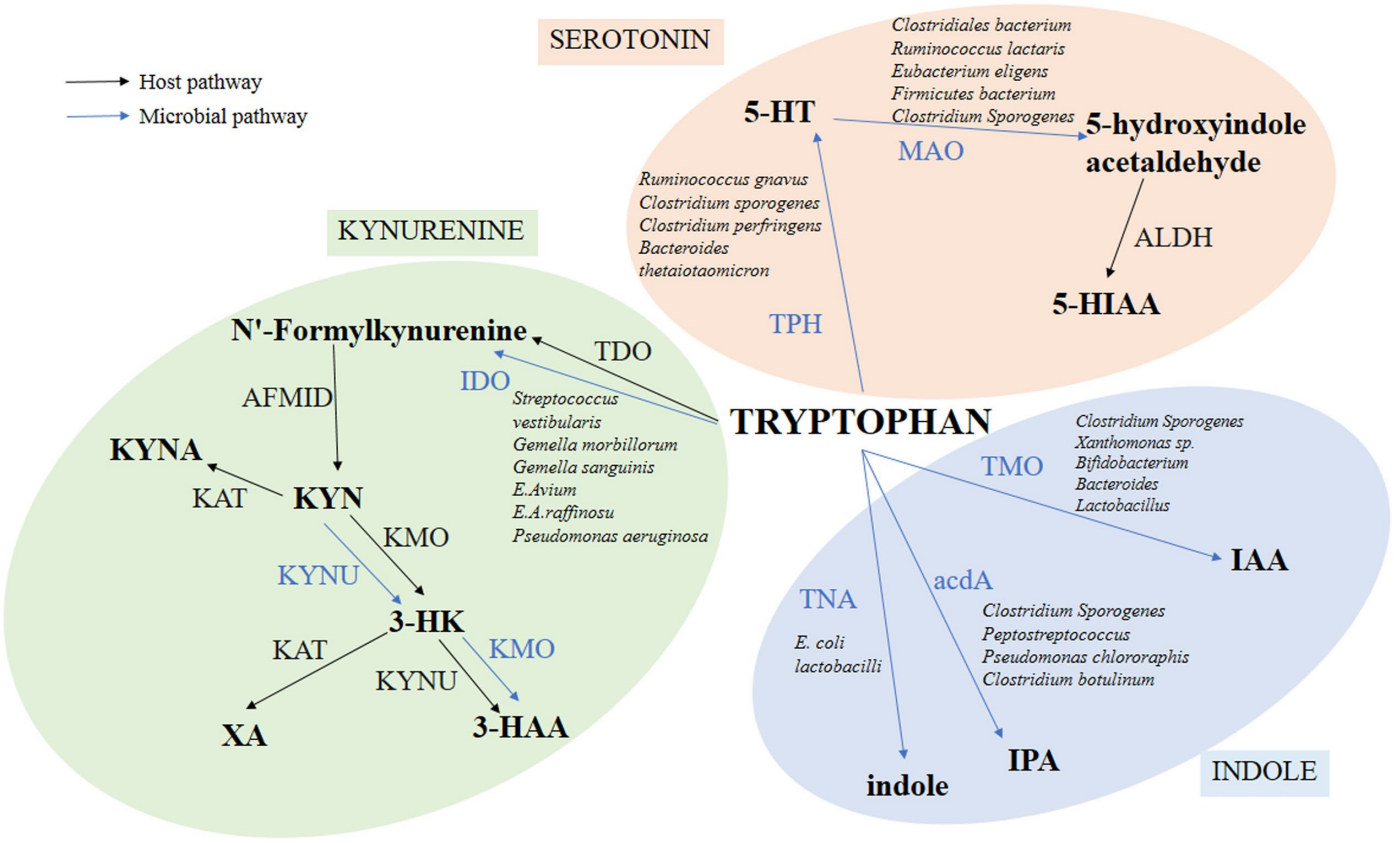
Pathways of TRP metabolism through 5-HT, KYN, and indole. The figure shows only the major metabolic processes and products, omitting some intermediate processes. Among them, the intestinal microbiota directly converts TRP into many kinds of bioactive molecules to execute multifold functions for intestine health. Blue lines indicate pathways for TRP metabolite in bacteria. Black lines indicate the TRP metabolite pathways in the host.

As shown in Figure 1, TRP has three metabolic pathways in the human body, including the KYN, 5-HT, and indole pathways (Platten et al., 2019; Modoux et al., 2021).

To screen out bacteria that can metabolize TRP, we cultured the 11 strains of bacteria (*Enterobacter hormaechei*, *Enterobacter cloacae*, *Klebsiella pneumoniae*, *Escherichia fergusonii*, *Weissella confusa*, *Klebsiella michiganensis*, *Shigella flexneri*, *Shigella sonnei*, *Klebsiella singaporensis*, *Proteus columbae*, and *C. sporogenes)* for 24 h under the corresponding liquid culture conditions. The supernatant was taken after centrifugation (8,000 × *g*, 4°C, 5 min), and the concentrations of IPA, 5-HT, 5-HIAA, and KYN in the bacterial suspension were detected according to the following LC–MS/MS detection method.

### Medium preparation and *Clostridium sporogenes* culturing

All operations were carried out under aseptic conditions. RCM medium was prepared in a 10 ml penicillin vial and sterilized at 121°C for 20 min. After purchasing the *C. sporogenes* strain from the supplier, we opened the ampoule containing the bacterial freeze-dried powder and strictly sterilized its outer packaging. Then, the freeze-dried powder was immediately mixed with an appropriate amount of normal saline. The *C. sporogenes* suspension was pumped into the penicillin vial and mixed well. The *C. sporogenes* was cultured in a shaker for 18–24 h at a speed of 200 × rpm, and the temperature was kept at 37°C. After the bacteria grew to obvious turbidity, 0.1 ml of bacterial liquid was absorbed and inoculated into the liquid RCM, and re-cultured for 24–48 h in an anaerobic environment at 37°C. The bacteria were stored in a 12 mm × 32 mm penicillin vial at −80°C and sealed with 25% (*v/v*) glycerin prepared in an anaerobic environment to ensure hypoxic conditions during long-term storage.

### Growth curves of *Clostridium sporogenes*

Based on the above results, we selected *C. sporogenes* as the subsequent drug screening model for further research. In addition, our previous study and the literature have reported that the abundance of *C. sporogenes* are different between CHD patients and healthy people (Jie et al., 2017; Liu et al., 2019). After *C. sporogenes* was recovered and subcultured, 400 μl of bacterial suspension was added into 6 ml of RCM medium, and three parallel groups were set for continuous culture for 24 h at 37°C. Samples were taken every 2 h for the first 12 h and every 4 h for the last 12 h (samples were taken at 2, 4, 6, 8, 10, 12, 16, 20, and 24 h). After mixing, 200 μl of samples was taken, and the OD_600_ was recorded with a microplate reader. The growth curve was drawn taking time as the abscissa and OD value as the ordinate.

### LC–MS/MS analysis

#### Chromatographic condition

The mobile phase (A) of liquid chromatography (LC-20A, Shimadzu, Japan) was 55% ultra-pure water containing 0.2% formic acid and 5 mM ammonium acetate. The mobile phase (B) was 55% methanol. The flow rate, column temperature, and chromatographic column were 0.3 ml/min, 35°C, and Luna® 3 μm HILIC 200 Å (Agilent Technologies, Santa Clara, CA, United States), respectively. The injection volume was 3 μl, and the analysis time was 4 min. Peak assignments in samples were made based on comparisons of retention times, and accurate mass-to-charge ratios from authentic standards were analyzed under identical conditions.

#### Mass spectrometry condition

The AB Sciex API4000 QTrap triple quadrupole mass spectrometer was used to scan in the multireaction detection (MRM) mode by using the electrospray ionization (ESI) and positive ion source. The basic mass spectrum parameters were as follows: ESI voltage, 5,500 V; air curtain air pressure (CUR), 25 psi; atomizing gas pressure (GS1), 50 psi; auxiliary gas pressure (GS2), 50 psi; and ion source temperature (TEM), 550°C. Peak areas were normalized using the internal standard, and concentrations were determined by comparison to calibration curves prepared from the dilution series of authentic standards for each compound.

### Preparation of standard solution and internal standard solution

The 5-HT-d4 was dissolved in acetonitrile to prepare 1 μg/ml of internal standard precipitation solution. Then, appropriate amounts of TRP, KYN, IPA, IAA, 3-HK, 3-HAA, KYNA, XA, 5-HT, and 5-HIAA standards were accurately weighed and used to prepare the standard mother liquor with 55% methanol. All standard solutions were stored at −80°C. Before use, an appropriate amount of mother liquor of the standard substance was collected and used to prepare stock solutions with acetonitrile (i.e., 100,000, 500, 100,000, 2000, 2000, 3,000, 500, 2000, 5,000, and 5,000 ng/μl).

### Pretreatment of bacterial sample

The blank culture medium should be pretreated first given the interference in the bacterial culture medium. The pretreatment was conducted by adding two times activated carbon following the volume of the blank culture medium. After vortex mixing for 48 h, the supernatant was centrifuged at 10,000 × rpm for 10 min. The supernatant was collected and filtered with a 0.45 μm microporous membrane. Similarly, bacterial samples were pretreated. After centrifugation (8,000 × rpm, 4°C, 5 min), 1 ml of supernatant was collected for subsequent operation. To precipitate proteins, the blank culture medium (50 μl) and bacterial samples (50 μl) were collected and added with 450 μl of internal standard precipitation solution. Then, the solution was allowed to stand at −20°C for 20 min. Subsequently, it was clarified by centrifugation (12,000 × rpm, 4°C, 20 min), and 100 μl of supernatant of the blank sample, bacterial sample, and mixed standard solution was separately injected into 96-well plates for testing. Moreover, the LC–MS/MS detection method was validated by the methodology. The method was adapted from a previously published method (Chen et al., 2019; Wicha-Komsta et al., 2020).

### Model optimization

In this study, a single-factor experiment and orthogonal experiment were used to analyze the optimal fermentation conditions of the model and to make the obtained drug screening model stable and reliable. We investigated the effects of the initial pH value, fermentation time, temperature, and inoculum amount on the total TRP concentration of *C. sporogenes*.

We inoculated 1% bacterial solution into an anaerobic flask containing sterilized RCM (pH = 6.8) and incubated it at 37°C. The LC–MS/MS technology was used to determine the total TRP concentration in the culture medium after 0, 2, 4, 6, 8, 10, 12, 14, 18, 20, 22, 24, 28, and 32 h, and the TRP concentration–time curve was drawn. Next, two of the factors [i.e., culture temperature (37°C), initial pH of culture medium (6.8), and inoculum quantity (4%)], were kept unchanged, whereas another factor was altered. Different gradients were set for each factor: culture temperature (25, 30, 37, 40, and 44°C), initial pH of the medium (4.8, 5.8, 6.8, 7.8, and 8.8), and inoculum volume ratio (*v/v*; 0.5, 1, 2, 4, 6, and 8%). Under the condition of 38 g/l RCM concentration, we changed one factor to investigate the effect of the factor on the total TRP level and performed culturing for 20 h. After the culture, the contents of TRP in the culture medium were determined using LC–MS/MS, and three parallel controls were set in each group to investigate the effects of different culture conditions on the level of TRP.

Based on the single-factor experiment, the inoculum amount, fermentation medium initial pH, and temperature were selected as three factors of the orthogonal experiment, and three levels were selected for each factor, as shown in Supplementary Table S7. Orthogonal table L9 (3^3^) was used for an orthogonal experiment with three factors and three levels. The optimum conditions were determined with LC–MS/MS to measure the concentration of TRP.

### Drug intervention experiment

In this part, we used a library of compounds, including IBA to intervene in this model. After the bacteria were resuscitated, 50 μl of bacterial solution was collected and inoculated into a 10 ml penicillin vial containing 8 ml of liquid RCM, and cultured for 36 h for activation. The bacteria were subcultured three times, and the bacterial solution was inoculated into a vial with 9.6 ml of sterilized liquid RCM by using a sterile syringe at an inoculum amount of 2% (*v/v*). The solution was cultured in an incubator maintained at 37°C. Three concentration gradients (20, 50, and 100 μM) were prepared for each group, and every concentration was prepared in triplicate. When the bacterial solution was cultured to the eighth hour (the bacteria were in the logarithmic growth period), 200 μl of drug sample solution was collected using a sterile syringe. Subsequently, the drug sample solution was injected into the medium, mixed uniformly, and incubated at 37°C for another 12 h. At the 20th hour, bacteria were collected (the bacterial growth was in the plateau period), and 1 ml of samples was transferred into the centrifuge tube. The supernatant was centrifuged at 12,000 × g for 10 min to obtain the bacterial metabolic components to be measured. The blank control group contained the only medium without bacteria added, the bacterial control group was added with the same amount of medium and DMSO, the drug group was added with an equal amount of medium and drugs dissolved in DMSO, and three parallels were set in each group.

### Statistical analysis

Data analysis was conducted by one-way ANOVA, and the statistical differences among groups were compared using Student’s t-test. Statistical significance was considered at *p* < 0.05 (^*^p < 0.05, ^**^p < 0.01, ^***^p < 0.001, ^****^p < 0.0001 vs. control group). GraphPad Prism version 8.0 (San Diego, CA, United States) was used for all statistical analyses.

## Results

### Screening of model bacteria

**Figure 2:**
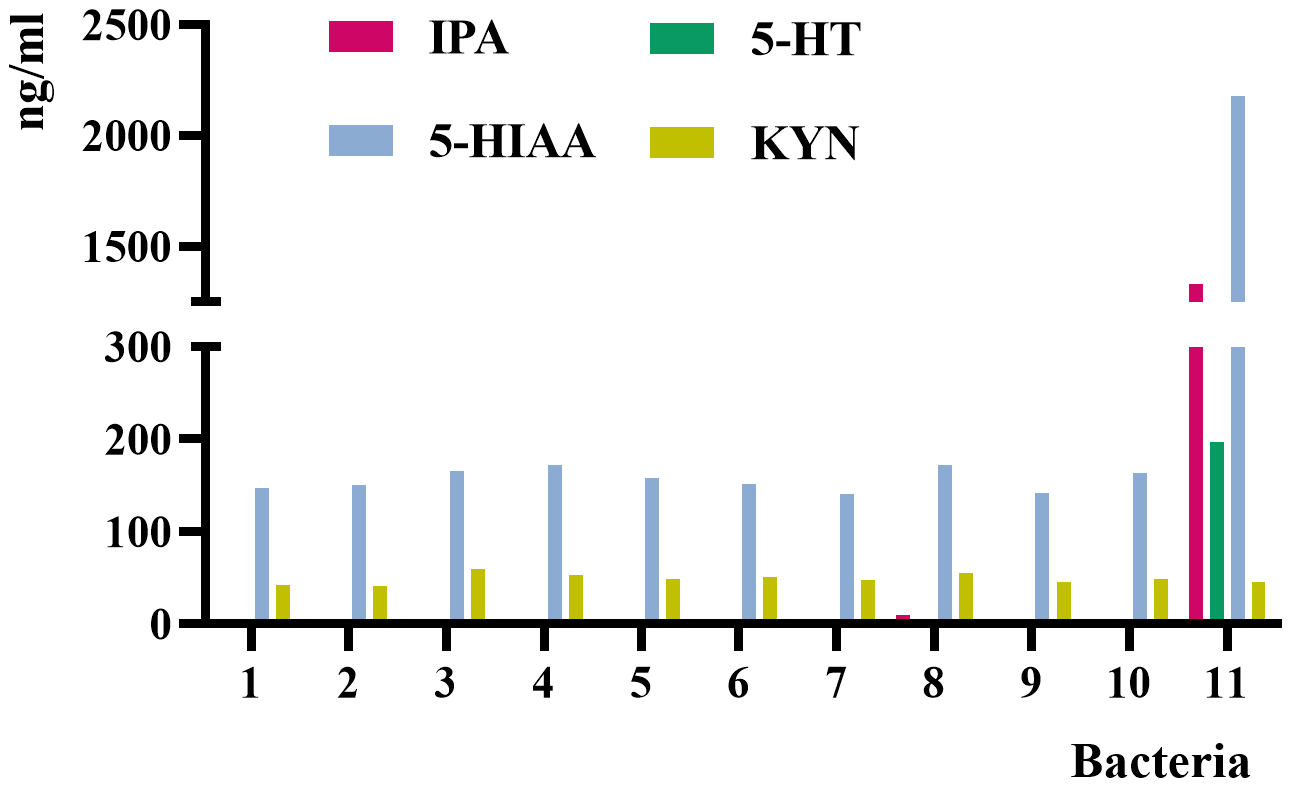
Concentrations of IPA, 5-HT, 5-HIAA, and KYN in the culture medium of 11 bacterial strains. 1, *Enterobacter hormaechei*; 2, *Enterobacter cloacae*; 3, *Klebsiella pneumoniae*; 4, *Escherichia fergusonii*; 5, *Weissella confusa*; 6, *Klebsiella michiganensis*; 7, *Shigella flexneri*; 8, *Shigella sonnei*; 9, *Klebsiella singaporensis*; 10, *Proteus columbae*; 11, *Clostridium sporogenes*.

Eleven individual isolates were screened from the feces of patients with CHD. The *C. sporogenes* culture showed highly significant levels of IPA, 5-HT, and 5-HIAA compared with other ten bacterial cultures, and all these bacterial cultures showed very low levels of KYN (Figure 2). Therefore, *C. sporogenes* was selected as the model bacterium in our subsequent evaluation experiments.

### Growth curves of *Clostridium sporogenes*

**Figure 3:**
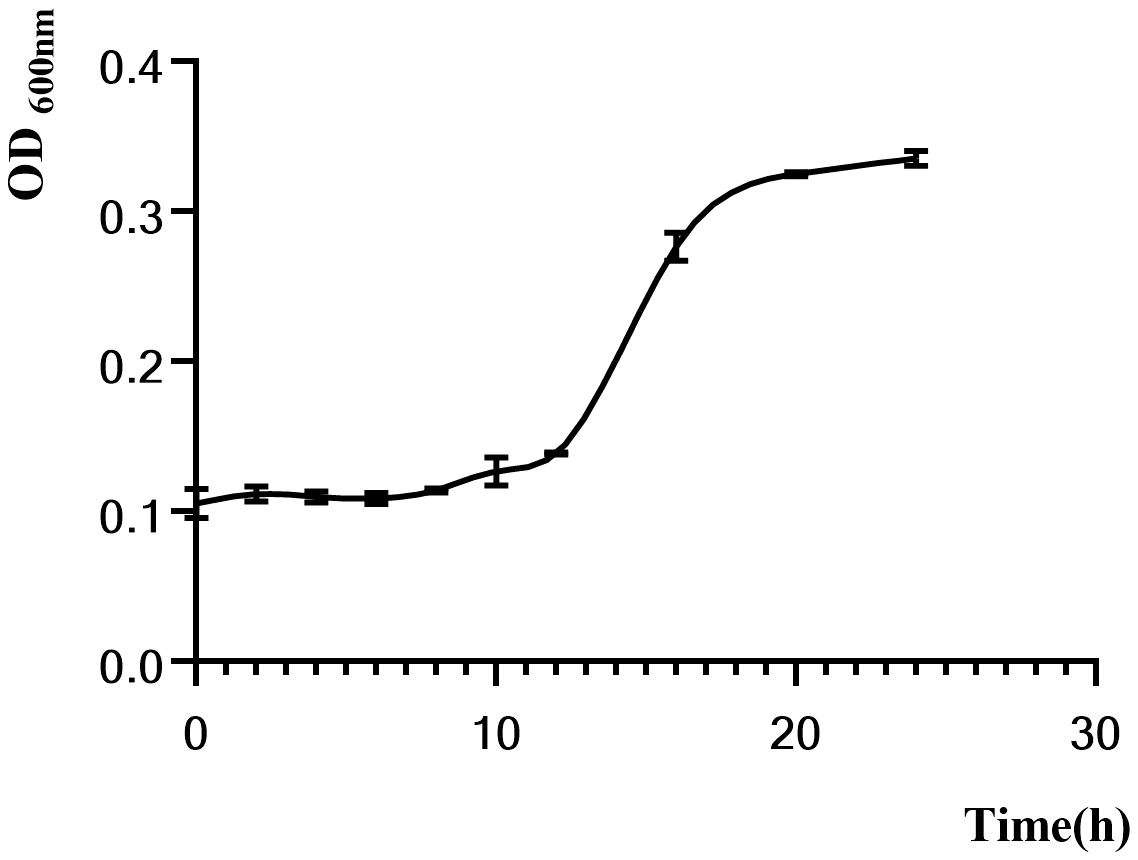
Growth curves of *C. sporogenes.*

Figure 3 shows that *C. sporogenes* was in the lag phase for the first 12 h and in the logarithmic phase for 12–16 h with a rapid increase in OD_600_. After the 16th hour, the bacteria were in the stationary phase.

### Determination of TRP and its metabolites by LC–MS/MS

**Figure 4:**
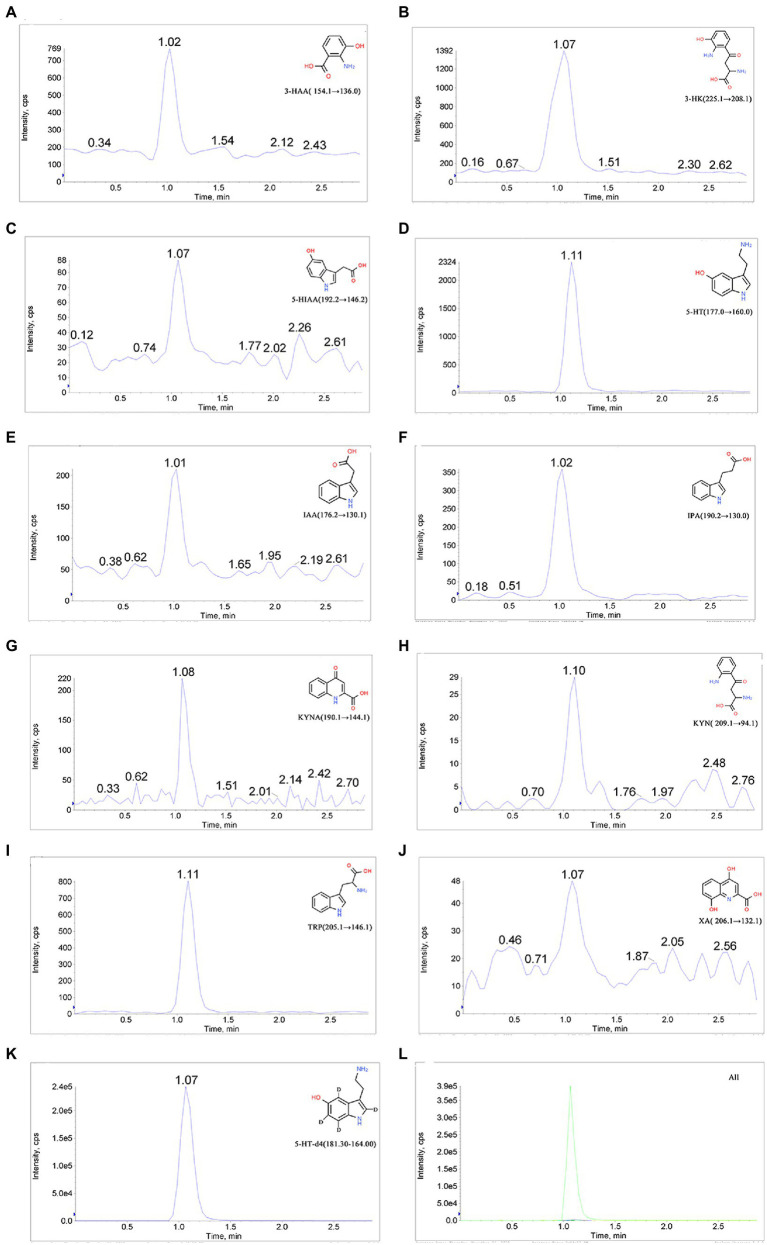
Chromatograms of 10 analytes and the internal standard. **(A)** 3-Hydroxyanthranilic acid (3-HAA), **(B)** 3-hydroxykynurenine (3-KH), **(C)** 5-hydroxyindole-3-acetic acid (5-HIAA), **(D)** 5-hydroxytryptamine (5-HT), **(E)** 3-indole-acetic acid (IAA), **(F)** indole-3-propionic acid (IPA), **(G)** kynurenic acid (KYNA), **(H)** kynurenine (KYN), **(I)** tryptophan (TRP), **(J)** xanthurenic acid (XA), **(K)** 5-hydroxytryptamine-d4 (5-HT-d4), and **(L)** total ion chromatogram.

We established the LC–MS/MS method for the detection of TRP, KYN, IPA, KYNA, 5-HT, 5-HIAA, 3-HK, 3-HAA, XA, and IAA in the RCM culture medium of *C. sporogenes*. The MRM mode monitoring reaction mass charge ratio (*m/z*) was as follows: 205.1 → 146.1 (TRP), 209.1 → 94.1 (KYN), 190.1 → 144.1 (KYNA), 154.1 → 136.0 (3-HAA), 206.1 → 132.1 (XA), 190.2 → 130.0 (IPA), 176.2 → 130.1 (IAA), 177.0 → 160.0 (5-HT), 225.1 → 208.1 (3-HK), 192.2 → 146.2 (5-HIAA), and 181.3 → 164.0 (5-HT-d4). The parameters for the MS/MS acquisition and extraction of compound-specific fragment ion traces are listed in Table 1. The chromatograms are shown in Figure 4.

**Table 1:** Optimal values of mass spectrometry parameters of TRP, its nine metabolites, and the internal standards.

Analyte	RT (min)	Precursor ion (*m/z*)	Product ion (*m/z*)	DP (V)	EP (V)	CE (eV)	CXP (V)
TRP	1.11	205.1	146.1	64.6	3.5	25.7	10.3
KYN	1.10	209.1	94.1	65.7	10.0	20.3	5.0
KYNA	1.08	190.1	144.1	75.0	10.0	30.0	10.0
3-HAA	1.02	154.1	136.0	37.0	10.0	16.7	10.0
XA	1.07	206.1	132.1	80.0	10.0	27.5	11.0
IPA	1.02	190.2	130.0	67.0	10.0	21.3	10.0
IAA	1.11	176.2	130.1	63.0	10.0	21.0	9.0
5-HT	1.11	177.0	160.0	45.0	3.50	19.0	13.4
3-HK	1.07	225.1	208.1	64.0	10.0	15.0	15.0
5-HIAA 5-HT-d4	1.07 1.07	192.2181.3	146.2164.0	84.0 40.2	10.0 4.0	25.1 14.0	11.7 8.50

TRP, Tryptophan; KYN, Kynurenine; KYNA, Kynurenic acid; 3-HAA, 3-Hydroxyanthranilic acid; XA, Xanthurenic acid; IPA, Indole-3-propionic acid; IAA, 3-Indoleacetic acid; 5-HT, 5-hydroxytryptamine; 3-HK, 3-Hydroxykynurenine; 5-HIAA, 5-Hydroxyindoles-3-acetic acid; 5-HT-d4, Internal standards.

### Optimum culture conditions of the model

**Figure 5:**
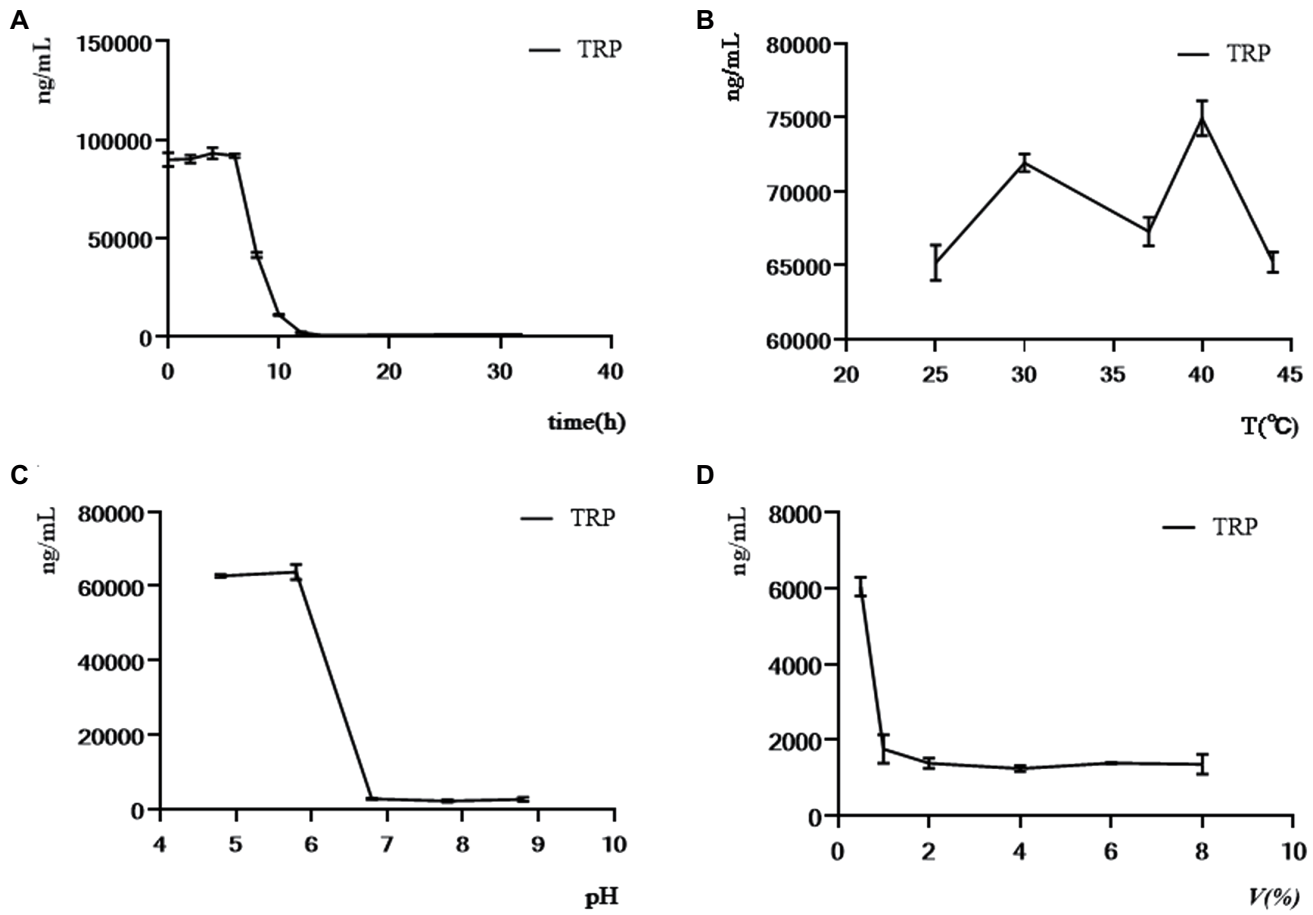
Effects of culture time, culture temperature, initial pH of the medium, and inoculum amount on the level of TRP in single-factor experiment. **(A)** The concentration of TRP was determined in 32 h while maintaining the culture temperature (37°C), initial pH of culture medium (6.8), and inoculum quantity (4%) constant. **(B)** The concentration of TRP at the 20th hour was determined while the initial pH of the medium (6.8) and inoculation amount (4%) remained constant in different temperatures (25, 30, 37, 40, and 44°C). **(C)** The concentration of TRP at the 20th hour was determined while the temperature (37°C) and inoculation amount (4%) remained constant in different initial pH of the medium (4.8, 5.8, 6.8, 7.8, and 8.8). **(D)** The concentration of TRP at the 20th hour was determined while the temperature (37°C) and initial pH of the medium (6.8) remained constant in different inoculation amounts (0.5, 1, 2, 4, 6, and 8%).

Four factors, including culture time, culture temperature, initial pH value of fermentation medium, and inoculum amount, were selected for the single-factor experiment. Considering that the influence of *C. sporogenes* on the production of TRP metabolites was studied, we detected the changes in total TRP levels of *C. sporogenes* with these four factors. The amount of TRP was almost unchanged within 0–6 h, decreased sharply within 6–12 h, and gradually stabilized (Figure 5A). Therefore, we chose to give the drug intervention at the eighth hour (when the TRP metabolism was in the logarithmic growth stage) and performed the sample detection at the 20th hour (when the TRP metabolism was in the stationary stage). The effects of the initial pH value and the inoculum amount on the TRP metabolites of *C. sporogenes* were shown as the minimum yield at 37°C, pH 6.8, and 2% inoculum amount (Figures 5B–D).

Based on the single-factor experiment, the L9 (3^3^) orthogonal experiment was used to optimize the conditions of TRP metabolites produced by cultured *C. sporogenes*. The design scheme and the results of the orthogonal experiment are shown in Table 2. K1, K2, and K3 were the sum of indicators at each level of each factor. K1 represents the sum of the values of the test indicators corresponding to one level, and so on. Ki = K1/3, Kii = K2/3, and Kiii = K3/3. R was the range, which was the difference between the maximum average TRP concentration minus the minimum average TRP concentration. The higher the R-value, the greater the influence of this factor on the concentration of TRP. Overall, with the concentration of TRP as the index, all the factors of primary and secondary order were determined through orthogonal experiment and extremum difference analysis: C-temperature > B-fermentation medium initial pH > A-inoculum amount. The optimal combination was A1B3C3. Our results revealed that the comprehensive ability of *C. sporogenes* to metabolize TRP was the best in the following condition: 40°C, initial pH = 7.8 of the fermentation medium, and 2% of inoculum amount. However, considering that the intestinal temperature of the human body was maintained at about 37°C under normal conditions, 37°C was still selected as the fermentation temperature to simulate the intestinal temperature. When the inoculation amount increased from 2%, the consumption of TRP gradually decreased, which can be explained by the fact that the high inoculation amount reduced the substrate used for the growth of bacteria. Hence, the optimal inoculation amount was determined to be 2%. In the orthogonal experiment, TRP’s consumption reached the maximum when the temperature was 40°C and the pH was 7.8. However, the pH of the small intestine is about 6.8 under normal conditions, and univariate results showed that TRP’s consumption is maximum at pH 6.8. Therefore, the pH of the medium was determined to be 6.8.

**Table 2:** Orthogonal experimental design.

NO.	Factor			
	A: Inoculation amount v/v%	B: pH	C: Temperature °C	TRP concentration ng/ml
1	2	5.8	30	15.5
2	2	6.8	37	9.54
3	2	7.8	40	2.71
4	4	5.8	37	12.7
5	4	6.8	40	6.36
6	4	7.8	30	12.1
7	6	5.8	40	9.67
8	6	6.8	30	14.3
9	6	7.8	37	11.3
K1	27.75	37.87	41.9	
K2	31.16	30.2	33.54	
K3	35.27	26.11	18.74	
Ki	9.25	12.62	13.97	
Kii	10.37	10.07	11.18	
Kiii	11.76	8.70	6.25	
R	2.51	3.92	7.72	

In summary, the optimal experimental conditions for the model were determined using the single-factor and orthogonal experiments. The last volume of the anaerobic bottle, sampling time, initial pH, inoculum amount, and culture temperature were 10 ml, 20 h, 6.8, 2%, and 37°C, respectively.

### Effects of *Clostridium sporogenes* on TRP metabolites

**Figure 6:**
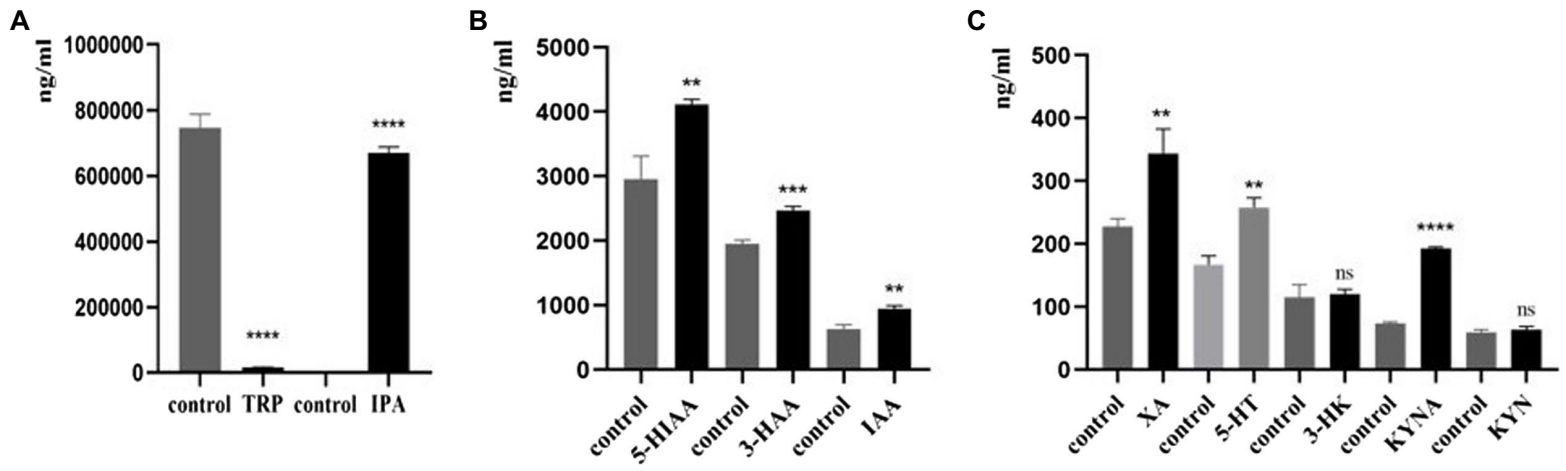
Effects of *C. sporogenes* on TRP metabolites. Effects of *C. sporogenes* on **(A)** TRP and IPA; (**B**) 5-HIAA, 3-HAA, and IAA; (**C**) XA, 5-HT, 3-HK, KYNA, and KYN (^*^p < 0.05, ^**^p < 0.01, ^***^p < 0.001, ^****^p < 0.0001 vs. control group).

In this study, we found that compared with the control group, *C. sporogenes* had significantly increased levels of IPA, 5-HIAA, 3-HAA, IAA, XA, 5-HT, and KYNA (Figures 6A–C); significantly reduced level of TRP (Figure 6A); and no effect on the generation of 3-HK and KYN (Figure 6C).

### Drug intervention experiment

**Figure 7:**
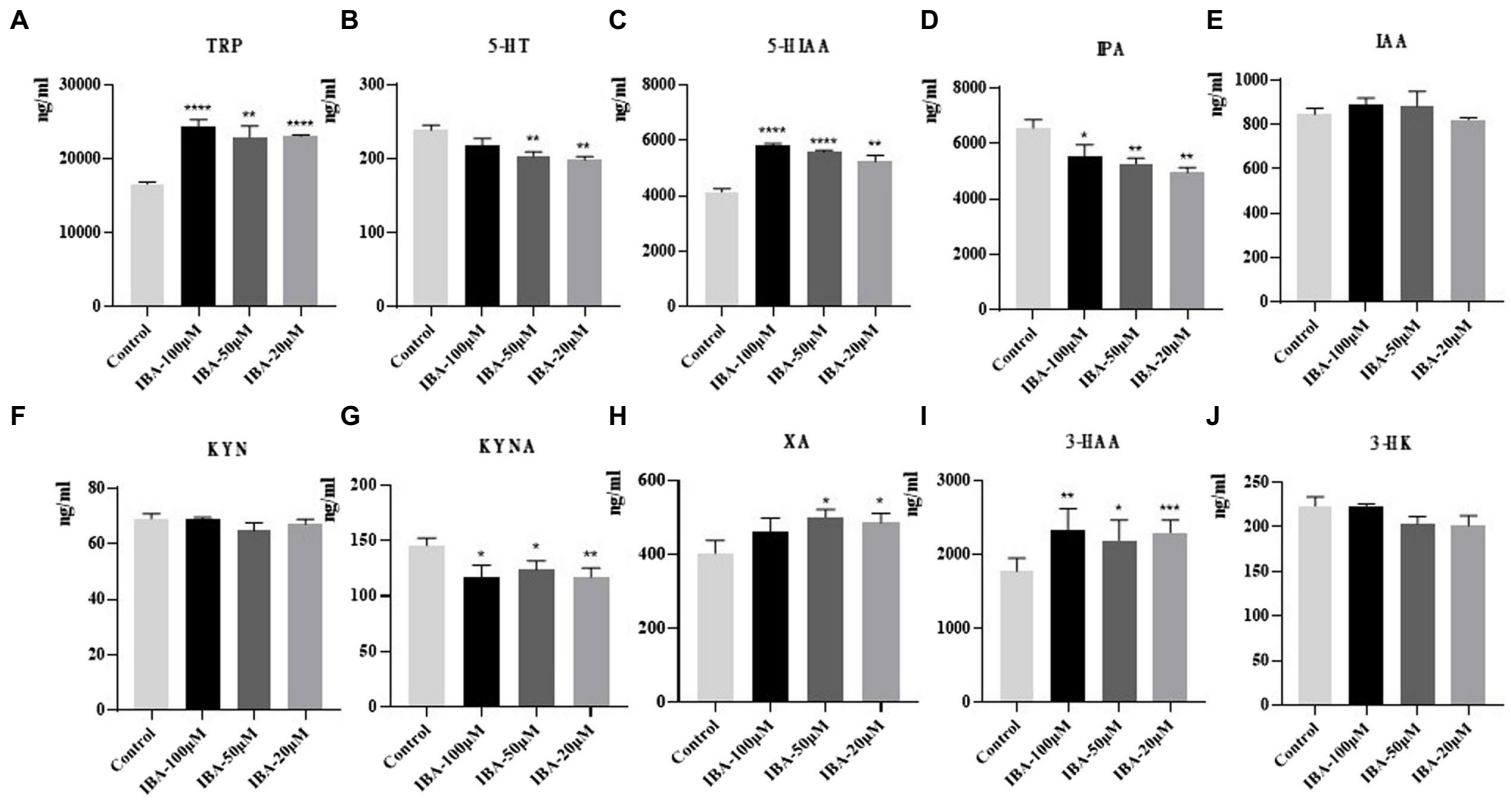
Results of drug intervention. Effects of IBA on **(A)** TRP, **(B)** 5-HT, **(C)** 5-HIAA, **(D)** IPA, **(E)** IAA, **(F)** KYN, **(G)** KYNA, **(H)** XA, **(I)** 3-HAA, and **(J**) 3-HK in *C. sporogenes*. IBA, indole-3-butyric acid. Three concentration gradients were set for IBA: 100, 50, and 20 μM (^*^p < 0.05, ^**^p < 0.01, ^***^p < 0.001, ^****^p < 0.0001 vs. control group).

After the model was established, we screened the library of compounds in our laboratory, among which IBA showed the most significant effect. The comparison between the drug and the control (to counteract the effect of the DMSO) showed that the amount of TRP increased significantly when IBA was added to the system (Figure 7A). At the same time, IBA could significantly increase the amount of 5-HIAA, XA and 3-HAA, and a high concentration of IBA promoted the improvement of the effect (Figures 7C,H,I). Moreover, the IBA could reduce the amount of 5-HT, IPA and KYNA (Figures 7B,D,G) but had little effect on other TRP metabolites (Figures 7E,F,J). The IBA was speculated to prevent TRP metabolism, so TRP is significantly increased. Because that the decreases of TRP metabolism, its downstream IPA pathway also decreases. At the same time, due to the reduced metabolism of 5-HT pathway, its downstream 5-HIAA will accumulate and will increase. Moreover, the overall amount of TRP still increased because the increased amount of 5-HIAA was much more than the reduced amount of IPA. It was also found that XA and 3-HAA increased significantly, while the amount of KYNA decreased, indicating that the downstream metabolism of KYN reached balance, so KYN did not change significantly. Additionally, the effect of IBA on TRP depended on the concentration.

## Discussion

**Figure 8:**
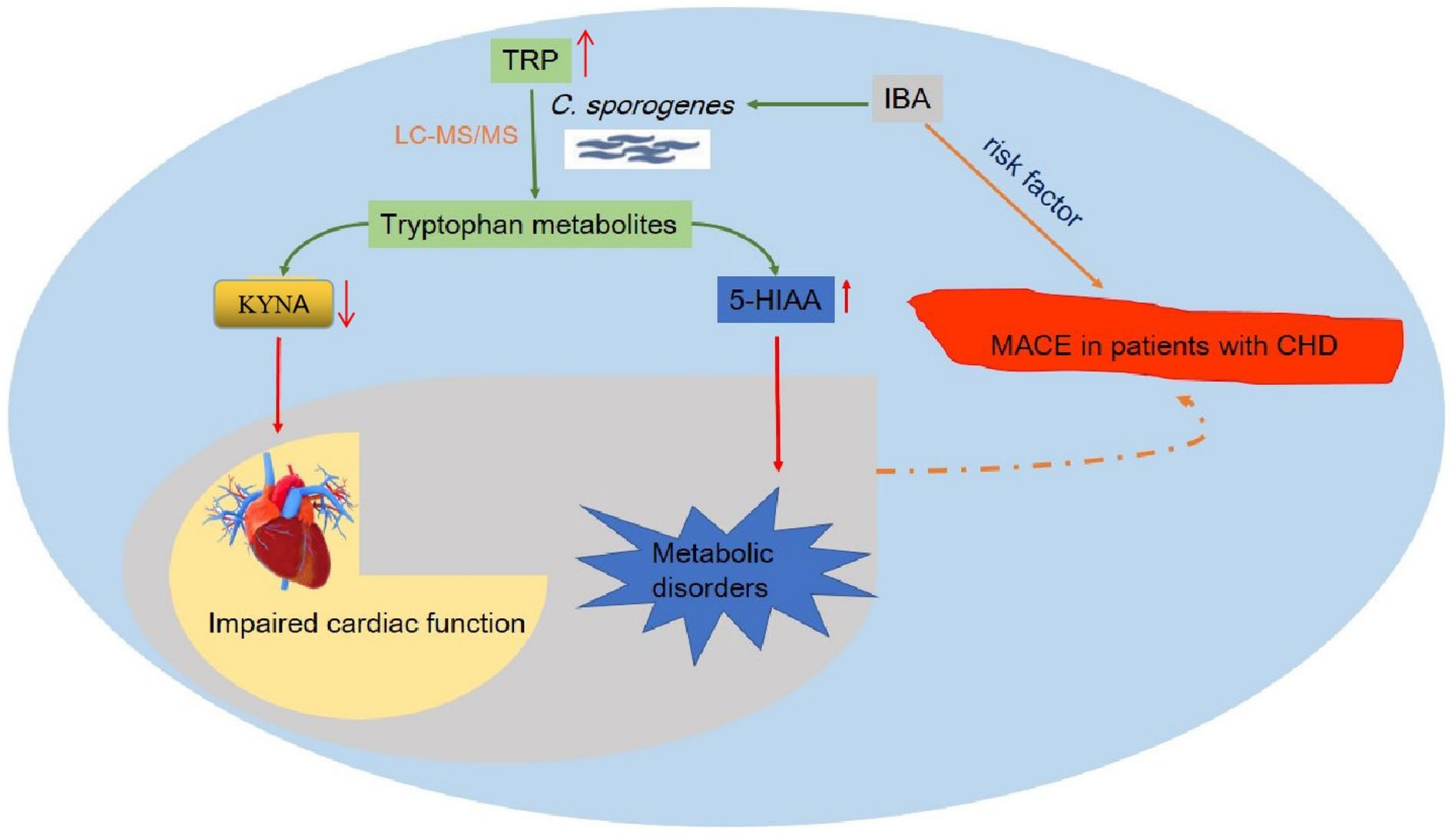
Overview of the possible mechanism of IBA affecting MACE in patients with coronary heart disease. This figure details a summary of the possible mechanism that IBA can increase the incidence of MACE in CHD patients by influencing the metabolism of TRP by *C. sporogenes* to produce IPA and 5-HIAA. TRP, tryptophan; IBA, indole-3-butyric acid; IPA, indole-3-propionic acid; 5-HIAA, 5-hydroxyindole-3-acetic acid; *C. sporogenes*, *Clostridium sporogenes*; MACE, major adverse cardiovascular events; CHD, coronary heart disease.

In this study, we established a novel drug screening model *in vitro* based on *C. sporogenes* and successfully applied a reliable and stable detection method to screen compounds for TRP and its metabolites in bacteria by using LC–MS/MS. In addition, we conducted experiments to investigate the effect of IBA on TRP metabolism. Our results showed that IBA could influence the levels of TRP, 5-HT, 5-HIAA, IPA, KYNA, XA and 3-HAA in the *in vitro* model of *C. sporogenes*. We summarized the possible mechanism that IBA could increase the incidence of MACE in CHD patients by influencing the metabolism of TRP by *C. sporogenes* (Figure 8).

The first goal of this work was to develop a drug screening model for specific metabolites produced by specific bacteria *in vitro*. As is well known, acting through metabolites is one of the main ways in which the gut microbiota interacts with the host (Lavelle and Sokol, 2020). The production of each gut microbiota-derived metabolite is mediated by one or several specific bacteria. For example, in the TRP pathway, the production of KYN can be mediated by *Streptococcus* and *Pseudomonas aeruginosa*. However, *E. coli* is the main intermediate of the metabolism of TRP to indole (Roager and Licht, 2018), and IAA is the only TRP metabolite in *Bifidobacteria* (Sakurai et al., 2019). *C. sporogenes* is one of the few bacteria that produce IPA (Skye et al., 2018). Thus, *in vitro* bacterial models that could produce specific metabolites for specific bacteria should be designed to screen what compounds could affect gut microbiota derivatives and which bacteria mediate this metabolite. To date, *in vivo* animal models and population cohort tests have been used to study the function and the dynamics of host-related microbiota (Li et al., 2018). On the other hand, for ethical, technical, regulatory, and cost reasons, *in vitro* methods are increasingly used as an alternative to *in vivo* experimentations. Although other studies have attempted to culture intestinal microbiota with complex methods, such as ChemoStats and microfluidic devices (McDonald et al., 2013; Ma et al., 2014), it is still not possible to determine which bacteria and compounds cause these metabolite changes by using these methods. In this study, an orthogonal experimental design method was adopted to optimize the conditions of the model, and the highly sensitive LC–MS/MS method was used to detect the level of TRP metabolites in the bacteria. This model can be used as a reference for the identification of specific bacteria and compounds that affect metabolites.

Herein, *C. sporogenes* was selected as the model bacteria. In our studies, 11 bacteria, which were mostly facultative anaerobes, including *Enterobacter hormaechei*, *Enterobacter cloacae*, *Klebsiella pneumoniae*, *Escherichia fergusonii*, *Weissella confusa*, *Klebsiella michiganensis*, *Shigella flexneri*, *Shigella sonnei*, *Klebsiella singaporensis*, *Proteus columbae*, and *C. sporogenes*, were isolated and identified from the feces of patients with CHD. After culturing for the same period, we detected the culture medium of these facultative anaerobes and *C. sporogenes*. We found that only *C. sporogenes* could produce 5-HIAA, 5-HT, and a large amount of IPA, whereas other bacteria could hardly produce IPA or could not reach the detection limit (Figure 2). This finding confirmed that IPA was indeed produced by *C. sporogenes* and not by other facultative anaerobes, which was consistent with other reports (Skye et al., 2018). Therefore, *C. sporogenes* was selected as the model bacteria for the *in vitro* drug screening. In our model, compared with the blank medium without *C. sporogenes*, the culture medium with *C. sporogenes* had significantly changed of TRP and its metabolites. This finding was also consistent with previous reports (Yano et al., 2015; Egamberdieva et al., 2017; Rosser et al., 2020). Notably, the generation of XA and KYNA has not been shown to be regulated by bacteria before. KYN may be converted to 3-HAA by kynureninase (KYNU) and KYNA and XA by kynurenine aminotransferases (KATI–KATIII; Schwarcz and Stone, 2017). In our study, it was found that XA and 3-HAA significantly increased, while the amount of KYNA decreased, indicating that the downstream metabolism of KYN reached a balance, so KYN did not change significantly. But the specific reason needs more experiments to prove in the future. Meanwhile, *C. sporogenes* could significantly promote the generation of XA and 3-HAA, indicating that *C. sporogenes* likely participated in their synthesis pathway, which might be a discovery.

Our results showed that IBA could significantly decrease the level of IPA and promote the metabolism of TRP into 5-HIAA in the *C. sporogenes* model. IPA is an example of the importance of bacterial involvement in the metabolism of TRP, and it was shown to be a metabolite that is absent in germ-free mice but is reintroduced following colonization with *C. sporogenes* (Wikoff et al., 2009). Our previous work and other reports have shown that the increased TRP metabolism toward IPA has a protective effect on the heart, which is beneficial to improve the survival prognosis of patients (Elmariah et al., 2018; Wang et al., 2021). 5-HT is a biogenic monoamine that plays various roles in metabolic homeostasis (Yano et al., 2015), and 5-HIAA is the end product of serotonin metabolism. Other results from human data also demonstrate that plasma levels of 5-HIAA are elevated in patients with obesity, dyslipidemia, and glucose metabolism disorders (Fukui et al., 2012; Agus et al., 2021). Increased 5-HIAA is also associated with high-sensitivity C-reactive protein, a marker of chronic low-grade inflammation underlying metabolic syndrome (Afarideh et al., 2015). In short, IPA and 5-HIAA are strongly associated with cardiovascular disease. Therefore, we proposed a bold conjecture that IBA may be a potential therapeutic intervention strategy for targeting TRP metabolic pathway by increasing the production of TRP and promoting the metabolism of TRP to 5-HIAA and inhibiting the production of KYNA. However, the specific mechanism of IBA affecting TRP metabolism through *C. sporogenes* is still unclear, which is also a problem to be solved in our follow-up work.

Although our research has made some achievements, there are still some limitations in this study. First, we have successfully developed an *in vitro* drug screening model for specific bacteria-specific metabolites, but this model is not yet capable of high-throughput screening. Second, there are many enzymes involved in TRP metabolism. We did not explore whether drugs regulate related metabolic enzymes and their mechanisms. Third, the gut microbiota is a very large community, and only *C. sporogenes* was studied in this work. Thus, further studies on animals and cells are needed to verify our speculation, to determine whether IBA can be used as a marker for the poor prognosis of CHD, and to clarify its mechanism of action on CHD through the gut microbiota.

In conclusion, our study successfully established a novel *in vitro* drug screening model for intestinal bacteria, providing an alternative method for identifying specific bacteria and compounds that affect metabolites. We aim to explore how metabolize TRP into beneficial metabolites against CHD or prevent from producing harmful metabolites by the *in vitro* drug screening model. As mentioned earlier, for patients with CHD, increased TRP is beneficial, while increased KYN is harmful. Our results show that IBA can significantly increase TRP and 5-HIAA, which are beneficial to CHD. At the same time, IBA can markedly increase XA and 3-HAA, while KYNA can dramatically decrease, so that the downstream of KYN can be maintained unchanged after reaching a balance. Logically, KYN should also be increased if TRP is significantly increased, but IBA is used to prevent this increase, thus satisfying the purpose of our study. Our research also demonstrated that it is possible to screen beneficial microbes from gut microbes to modulate coronary heart disease. The metabolite targets of other diseases can also be used to build models to screen drug compounds, which has great application prospects.

## Data availability statement

The original contributions presented in the study are included in the article/Supplementary material, further inquiries can be directed to the corresponding author.

## Author contributions

XXT, YYW, CD and XHZ performed the research. YL, JBZ, and WHL revised the manuscript. SYZ, LXC, and SLZ designed the research. All authors contributed to the article and approved the submitted version.

## Funding

This study was funded by grants from the National Nature Science Foundation of China (no. 81872934 and 81673514), the Key research and development program of Guangdong Province, China (no. 2019B020229003), Science and Technology Development Projects of Guangdong Province, China (no. 2017B0303314041), Science and Technology Planning Project of Guangdong Province of China (no. 2019A050510025), and Guangdong Provincial People’s Hospital Clinical Research Fund (Y012018085).

## Conflict of interest

The authors declare that the research was conducted in the absence of any commercial or financial relationships that could be construed as a potential conflict of interest.

## Publisher’s note

All claims expressed in this article are solely those of the authors and do not necessarily represent those of their affiliated organizations, or those of the publisher, the editors and the reviewers. Any product that may be evaluated in this article, or claim that may be made by its manufacturer, is not guaranteed or endorsed by the publisher.

## Abbreviations

KYN: Kynurenine
KYNA: Kynurenic Acid
XA: Xanthurenic acid
3-HK: 3-Hydroxykynurenine
3-HAA: 3-Hydroxyanthranilic acid
IAA: 3-Indole-acetic acid
IPA: Indole-3-propionic acid
5-HT: 5-hydroxytryptamine
5-HIAA: 5-Hydroxyindole-3-acetic acid
IDO: Indoleamine-2,3-dioxygenase
TDO: Tryptophan-2,3-dioxygenase
AFMID: Kynurenine formamidase
KMO: Kynurenine-3-monooxygenase
KYNU: Kynureninase
KAT: Kynurenine aminotransferase
TNA: Tryptophanase
ACDA: Acyl-CoA dehydrogenase
TPH: Tryptophan hydroxylase
MAO: Monoamine oxidase
